# The Modulation of Cell Plasticity by Budesonide: Beyond the Metabolic and Anti-Inflammatory Actions of Glucocorticoids

**DOI:** 10.3390/pharmaceutics17040504

**Published:** 2025-04-11

**Authors:** Eduardo Jorge Patriarca, Cristina D’Aniello, Dario De Cesare, Gilda Cobellis, Gabriella Minchiotti

**Affiliations:** 1 Stem Cell Fate Laboratory, Institute of Genetics and Biophysics “A. Buzzati Traverso”, National Research Council, 80131 Naples, Italy; cristina.daniello@igb.cnr.it (C.D.); dario.decesare@igb.cnr.it (D.D.C.); 2 Department of Precision Medicine, University of Campania Luigi Vanvitelli, 80138 Naples, Italy; gilda.cobellis2@unicampania.it

**Keywords:** budesonide, stem cells, gastruloids, cancer cells, metastasis, SARS-CoV-2

## Abstract

The synthetic cortisol analog budesonide (BUD) is an essential drug employed to manage chronic inflammatory diseases in humans, mainly those involving gastroenteric and airway mucosa, such as rhinitis, laryngitis, bronchitis, esophagitis, gastritis, and colitis, with high levels of success. As a glucocorticoid, BUD prevents the expression of pro-inflammatory cytokines/chemokines and the recruitment of immune cells into the inflamed mucosa. However, emerging evidence indicates that BUD, unlike classical glucocorticoids, is also a potent modulator of stem and cancer cell behavior/plasticity. Certainly, BUD stabilizes cell–cell adhesions, preventing embryonic stem cell differentiation and inhibiting the development of 3D gastruloids. In addition, BUD inhibits the motile/invasive propensity of different cancer cells, including breast, lung, and pancreatic cancer. Finally, it prevents the infection of positive single-stranded human-infecting RNA viruses such as SARS-CoV-2. At a molecular level, BUD induces epigenetic changes and modifies the transcriptome of epithelial, stem, and cancer cells, providing molecular support to the immune cell-independent activity of BUD. Here, we performed an in-depth review of these unexpected activities of BUD, identified by unbiased drug screening programs, and we emphasize the molecular mechanisms modulated by this efficacious drug that deserve further research.

## 1. Introduction

**Figure 1:**
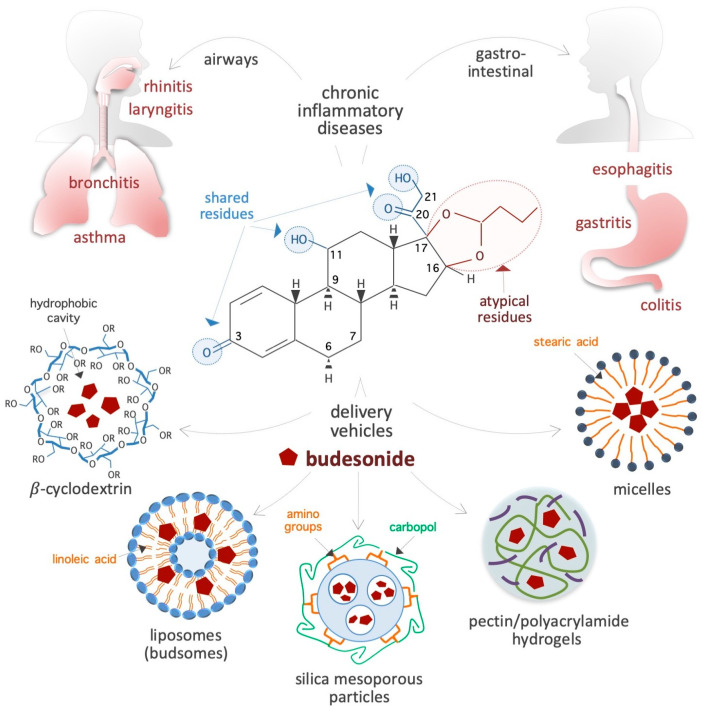
Schematic showing the chemical structure of the synthetic corticosteroid BUD, diseases treated with BUD, and vehicles for delivery. The residues shared by BUD, hydrocortisone, and classical corticosteroids are indicated by light blue circles, whereas the specific/atypical structural features of BUD are shown in red. The chronic IDs that affect the gastroenteric and airway mucosa and are usually treated with BUD are presented on top. Innovative BUD delivery vehicles are shown at the bottom. In the chemical structure of cyclodextrin, R = 2-hydroxypropyl or H.

Glucocorticoids (GCs) are a group of steroid hormones mainly produced by the adrenal glands that play important roles in stress response [1]. An important function of GCs is their anti-inflammatory action, through which they are involved in the regulation of immune and metabolic pathways. As a result of these functions, synthetic GCs were developed in the 1950s for systemic anti-inflammatory therapy [2]. Among the GCs that are currently in use, budesonide (BUD) is a steroidal anti-inflammatory drug that was first introduced in the early 1980s to treat bronchial asthma [3,4,5]. Since then, it has been extensively studied and applied for the treatment of a range of inflammatory conditions. Due to its proven effectiveness, safety, and affordability, the World Health Organization has listed BUD as an essential drug for the management of chronic inflammatory diseases (IDs), especially those affecting the respiratory system (e.g., rhinitis, laryngitis, and asthma) and gastrointestinal tract (e.g., esophagitis, gastritis, and colitis) (Figure 1). In addition to these therapeutic properties of BUD, which are well investigated, some recent evidence has emerged about the ability of this drug to also impact stem cell plasticity, cancer cell metastasis, and the replication of positive-strand RNA viruses. While the evidence is convincing, the known anti-inflammatory activities of BUD do not adequately explain these emerging biological effects. Given the potential of BUD for the treatment of cancer and viral infections, it is imperative to have a deeper understanding of its mechanisms, which would help to design treatment strategies involving BUD in the future. Therefore, this review delves in depth into the reported BUD actions—anti-inflammatory, pro-epithelization, pro-pluripotency, anti-plasticity, anti-metastatic, and anti-viral—and attempts to present a comprehensive picture of the wide-ranging therapeutic potential of BUD and the related mechanisms. This paper starts with a description of the physicochemical properties of BUD and then moves on to its mechanisms in mucosal inflammatory conditions, stem cell plasticity, cancer cell metastasis, and viral replication. Finally, we present our concluding remarks on the state of research so far and future prospects for research and the clinical application of therapeutic strategies with BUD.

## 2. Physicochemical Properties of Budesonide

### 2.1. Chemical Structure and Properties

BUD is a synthetic non-halogenated corticosteroid represented as (R,S)-16α,17α-butylidenedioxy-11β,21-dihydroxypregna-1,4-diene-3,20-dione (CAS 51333-22-3, CHEMBL1370, HMDB0015353, D00246, molecular formula: C_25_H_34_O_6_, molecular mass: 430.53 g/mol) (Figure 1). Regarding its chemical structure, BUD share three residues with cortisol (50-23-7, C_21_H_30_O_5_, molecular mass: 362.46 g/mol), namely, a ketone, a hydroxyl, and a 2-hydroxyacetyl residue located at positions 3, 11, and 17, respectively, of the fundamental four-ring steroid backbone (Figure 1). However, BUD differs from typical GCs (such as hydrocortisone, prednisolone, and dexamethasone) because of its pentacyclic structure (butylidene-bis-oxy) located at positions 16 and 17 of the four-ring steroid backbone (Figure 1). Consequently, BUD is less soluble in water (46 vs. 320 μg/mL) than hydrocortisone, has 200-fold more affinity for glucocorticoid receptors (GRs) than hydrocortisone [6], and exhibits potent GC activity and weak mineralocorticoid activity [7]. The BUD concentration in plasma and dried blood spots can be measured by using 3-keto-desogestrel or budesonide-d8 as an internal standard [8,9,10,11,12,13,14].

### 2.2. Bioavailability and Carriers for Delivery

Bioavailability: BUD is preferred to other corticosteroids due to its extensive (>80%) first-pass hepatic metabolism through cytochrome P450/CYP3A enzyme activity, the low potency of its derivative metabolites (16α-OH-prednisolone and 6β-OH-BUD) [15,16], and its function as a substrate of the drug efflux pump P-glycoprotein [17]. These peculiar features contribute to its low systemic bioavailability and minimal side effects that result from the suppression of the hypothalamic–pituitary–adrenal axis [18,19,20]. For instance, 6 h after the inhalation of a single dose (1600 μg) of BUD, the serum concentration reaches only 1 nM [21].

Carriers: BUD usually forms a complex with solubility enhancer molecules such as hydroxypropyl-β-cyclodextrin (a cone-shaped cyclic oligosaccharide) (Figure 1), which is able to host hydrophobic steroids [22,23,24,25]. Moreover, as shown in Figure 1, several innovative delivery carriers for pharmacological agents that could also be used for BUD administration are under development: for example, colloidal nanoliposomes (referred to as budsomes) with BUD linked to linoleic acid via a hydrolytic ester bond [26], nanosized micelles created by the conjugation of stearic acid with caffeic acid (an amphiphilic compound) [27], biodegradable pectin/polyacrylamide hydrogels [28], hyaluronic acid nanoparticles [29], polylactide-*co*-glycolide acid and dl-polylactide nano- and microparticles [30,31], pegylated liposomes [32], mesoporous silica nanoparticles [33], bile acid-based vesicles (bilosomes) and nanoparticles (biloparticles) [34], nanostructured diatomite biosilica microparticles [35], and mannosylated BUD palmitate nanoparticles [36]. BUD-loaded nanoparticles (diameter, 57 nm) incorporated into hydrogels and poloxamer hydrogels have been generated for the treatment of atopic dermatitis [37,38]. Capsules were designed to release BUD in the distal ileum/ileocecal region (targeted release formulation) for the treatment of IgA nephropathy [39,40]. In addition, BUD containing suppositories produced by semi-solid extrusion-assisted 3D printing are now available for the treatment of colitis [41,42]. As a result of such advancements in the research on delivery agents and methods, it is expected that efficient BUD carrier systems will soon become available in the market.

### 2.3. Pharmacokinetic Parameters

A randomized double-blind trial performed on healthy subjects revealed that, after a single inhaled dose (320 μg, day 1) of BUD, the maximal plasma concentration (C_max_ = 459 pg/mL) was reached in less than 1 h (t_max_ = 0.3 h), and the elimination half-life (t_1/2_) of BUD was ~4.6 h [43]. Moreover, a trial designed to evaluate the systemic exposure of pediatric patients with eosinophilic esophagitis (EoE) to a single dose of an oral suspension of BUD (0.35–2 mg) in the morning reported comparable values between patients (C_max_ = 492 to 1019 pg/mL; t_max_ = 0.7 to 1.1 h; t_1/2_ = 3.3 to 3.5 h) [44]. Furthermore, a single dose (3 mg) of BUD in pediatric patients with Crohn’s disease revealed similar values (C_max_ = 1760 pg/mL; t_max_ = 4.5 h; t_1/2_ = 1.9 h) [45]. A relevant fraction (>80%) of BUD is converted in the liver into two major and inactive metabolites, namely, 6β-OH-BUD and 16α-OH-prednisolone, by the activity of the CYP3A (cytochrome P450, family 3, subfamily A) enzyme [46,47]. BUD conversion in the liver reduces systemic exposure and prevents any relevant BUD accumulation. The frequency of adverse drug reactions following BUD treatment is lower than those induced by conventional steroids [48], and the administration of BUD via inhalation further reduced the risk of systemic exposure. Indeed, when BUD is delivered directly into the lung (through inhalation or nebulization), its concentration in the plasma is lower than that after systemic administration (orally or through an injection). For instance, 90 min after a single inhalation of 1.6 mg of BUD, its concentration was 8-fold higher in lung tissue (2–9 nmol/kg) than in blood plasma (0.27–1.1 nmol/kg) [49]. However, when BUD is administered at high dosages (3 mg thrice daily for 1 week), there is a risk of reversible adrenal suppression (associated with reduced cortisol plasma levels) [45]. Thus, BUD appears to have a good safety profile based on the findings reported in the literature, and inhalation seems to be the most beneficial method of administration. Recently, however, a novel method to prepare a soluble BUD derivative, i.e., budesonide 21-phosphate (BUD-21P), has been described [50].

### 2.4. Molecular Targets and Mechanisms of Action

Similar to classical GCs, BUD binds the GR N3CR1, a member of the nuclear receptor superfamily of ligand-dependent transcription factors, and modifies the transcription profile of the target cells. For instance, in explants of human fetal lung tissue, BUD (30 nM, 4 days) was found to alter the expression of around 1000 genes associated with inflammatory pathways (e.g., interleukin 1β/IL1β, lipopolysaccharide), transforming growth factor-β1/TGFβ1 and the tumor necrosis factor/TNF [51]. Further, in airway smooth muscle (ASM) cells derived from asthma patients, BUD (100 nM, 24 h) was found to deregulate a large (>5 × 10^3^) set of genes involved in inflammatory/cytokine-related pathways, amino acid and lipid metabolism, focal adhesion, and gap junctions [52]. BUD was found to induce the production of cAMP in a GR-independent manner by activating the G protein-coupled receptor Gα in primary human ASM cells [52] and to inhibit both the histamine-induced contractions in guinea pig ASM cells [53] and the production of pro-inflammatory cytokines/chemokines from human lung macrophages activated by secretory phospholipases A(2)sPLA(2) [54]. BUD was found to inhibit Smoothened (Smo) ciliary translocation and Hedgehog signaling in NIH3T3 cells [55,56] and to promote in vitro myelination [57]. There is also some evidence of epigenetic mechanisms at play, as BUD induced DNA methylation in fibroblasts isolated from the hamster when it was administered at very high concentrations (50–70 μM) [58]. In addition, BUD also induces the re-epithelization of damaged mucosa and inhibits the pluripotency exit in stem cells, metastatic progression in cancer cells, and RNA virus invasion/replication, which are exhaustively discussed later in this review. However, it should be noted that there are still many unanswered questions regarding the underlying molecular mechanisms involved in BUD actions.

## 3. Budesonide-Mediated Mucosal Re-Epithelialization

**Figure 2:**
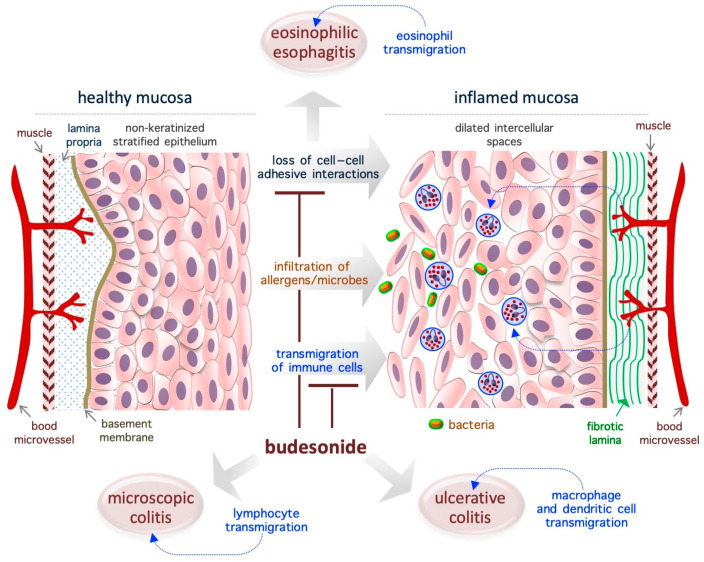
Schematic representation of the re-epithelialization actions of BUD in gastroenteric inflammatory diseases. The diagram is representative of eosinophilic esophagitis, but similar effects of BUD are described in ulcerative and microscopic colitis. The loss of cell–cell adhesive interactions reduces epithelial barrier activity and allows for the penetration of allergens, chemicals, and/or microorganisms, which is followed by the infiltration of immune cells and, eventually, inflammation. BUD suppresses mucosa inflammation by promoting cell–cell adhesion and inhibiting immune cell transmigration.

In patients with chronic IDs, the normal tightly packed structure of the mucosa is turned into a disorganized (relaxed and loose) tissue that is invaded by immune cells such as lymphocytes, eosinophils, mast cells, neutrophils, and macrophages. It is widely accepted that BUD exerts a strong GR-dependent anti-inflammatory effect that inhibits the expression of inflammatory mediators (cytokines, eicosanoids, histamine, and leukotrienes) and the activation/migration of immune cells [18]. However, emerging evidence indicates that BUD also impacts epithelial cells at the molecular (in terms of gene expression profile and metabolism) and behavioral (in terms of growth rate and adhesive ability) level (Figure 2). The next few subsections present an in-depth evaluation of the effects of BUD on the mucosa of patients with IDs, with a focus on its re-epithelialization action.

### 3.1. Eosinophilic Esophagitis

EoE is a chronic T helper type 2-associated esophageal disorder with an estimated incidence of 1–20 new cases per 100,000 inhabitants per year and a male-to-female ratio of 3:1, and topical BUD treatment (oral suspension, 2 mg twice daily) is the preferred treatment [59,60,61,62,63,64,65,66]. Genetic traits and exposure to acid, food allergens, or aero-allergens are considered as the causative factors of EoE [67]. While eosinophils are typically absent in healthy esophageal mucosa (Figure 2), in the EoE mucosa, the eosinophils transmigrate across the non-keratinized stratified squamous epithelium and cause esophageal eosinophilia (threshold = 15 to 16 eosinophils/high-power field, ~0.3 mm^2^) [63,65]. Moreover, the normal mucosa is replaced by a hyperplastic epithelium with dilated intercellular spaces (causing impaired barrier function) and a fibrotic lamina propria [65] (Figure 2). Of note, in the EoE mucosa, nearly 5000 genes are deregulated, and a fraction of them are associated with epithelial function/integrity [65,68]. Specifically, EoE severity (based on the presence of a narrow-caliber esophagus and basal zone hyperplasia) is more closely associated with the deregulation of epithelial-related genes (e.g., *ACPP*, *CITED2*, *CTNNAL1*, *EML1*, *FLG*, *GRPEL2*, *MT1M*, *PNLIPPR3*, and *TSPAN12*) than with the degree of eosinophilic invasion [69]. In particular, a genome-wide association study revealed the presence of an association between the *DSG1* gene (coding for the desmosomal cadherin desmoglein-1, an intercellular adhesion molecule) and the risk of EoE. Accordingly, it has been shown that silencing of the *DSG1* gene is sufficient to induce EoE-like epithelial features in immortalized esophageal epithelial cells (that is, EPC2 cells) [70]. Thus, the pathogenesis of EoE appears to be closely related to disruption of the adhesive ability of epithelial cells in the mucosa, and, therefore, agents that target adhesion-related proteins might be ideal therapeutic agents. In the EoE mucosa, BUD has been found to alter the expression of epidermal cornifins (e.g., SPRR1A, 2A, 2B, and 2D) and transglutaminase enzymes (TGM1 and TGM3), which are potent modifiers of the pericellular matrix [71,72,73]. Further, BUD-induced EoE remission was associated with reestablishment of the normal epithelial phenotype [72,74]. Similarly, BUD was able to restore the barrier integrity in a cell model of eosinophilic chronic rhinitis with nasal polyps (eos-CRSwNP) treated with particular matter 2.5 (PM_2.5_) [75].

### 3.2. Ulcerative Colitis

Ulcerative colitis (UC) is a chronic idiopathic ID that affects the colon and is showing an increasing worldwide prevalence, with an incidence rate of 10–20 in every 100,000 individuals, a relapsing and remitting course, and a higher prevalence in women aged 20–30 years; BUD (9 mg once daily, 8 weeks) is typically used as a first-line therapy to treat mild/moderate UC [76,77,78]. UC is associated with the infiltration of macrophages and dendritic cells into the lamina propria. In a mouse model of colitis induced by dextran sulfate sodium, BUD was found to inhibit the expression of cytokines (e.g., myeloperoxidase/MPO and tumor necrosis factor/TNF-α) and inflammatory enzymes (e.g., cyclooxygenase-2/COX-2 and inducible nitric oxide synthase/iNOS) [27]. UC is usually associated with significant defects in the intestinal epithelium (reduction in the covering mucinous layer and tight junctions), but it not clear whether the observed epithelial alterations are the cause or consequence of UC [76]. BUD has been found to promote intestinal mucosa repair/mucosal healing in rat UC models (generated using the haptenizing agent oxazolone) [79], as well as in patients with UC [80,81].

### 3.3. Microscopic Colitis

Microscopic colitis (MC) is a persistent intestinal disorder that affects the function of the colon, is more prevalent in women than in men (with a female/male ratio of 2:1), has an incidence rate of 0.6–16 cases per 100,000 persons/year, and usually manifests in individuals aged 60–65 years [82,83,84,85]. Biopsy samples taken from various parts of the colon are necessary for the correct diagnosis of MC [85]. Two histologic subtypes of MC have been described: lymphocytic colitis, which is characterized by over 20 intraepithelial lymphocytes per 100 epithelial cells, and collagenous colitis, which is characterized by a similar increase in lymphoplasmacytic infiltration into the lamina propria and is associated with a thickened subepithelial collagen band (thickness, >10 µm) [86,87,88]. According to the results of several randomized controlled trials, BUD is an effective agent for the management of MC when it is administered at doses of 9 mg/day and 6 mg/day for the induction and maintenance of clinical remission, respectively [82,89,90]. With regard to the underlying mechanism, BUD treatment has been found to alter the expression of genes involved in collagen metabolism, extracellular matrix organization, cell–cell adhesion, and energy metabolism, and, most remarkably, to play a role in restoring epithelial architecture/function [91]. Moreover, the long-term use of BUD is well tolerated and has limited adverse effects, making this a viable option for MC treatment [90]. Thus, the re-epithelialization action of BUD appears in the context of EoE, UC, and MC.

### 3.4. Asthma

The administration of BUD via inhalation (180 to 360 μg via oral inhalation twice daily) has been used to treat mild-to-moderate persistent asthma from the early 1980s. In patients with asthma, the airway mucosa loses its integrity as a result of the induction of epithelial-to-mesenchymal transition (EMT) [92] and increased fibrosis (of the basement membrane) (for a review, see [93]). Of note, similar alterations in the epithelial structure are observed in children with asthma even before clinical manifestations of the disease are observed and in the absence of inflammation [94,95]. These observations support the idea that epithelial remodeling is a cause, and not a consequence, of chronic inflammation in patients with asthma. Importantly, and in relation to the mechanisms of BUD, the analysis of airway inflammatory diseases has shown that BUD has a similar re-epithelialization effect in these diseases.

## 4. Budesonide-Mediated Inhibition of Plasticity in Stem and Cancer Cells

**Figure 3:**
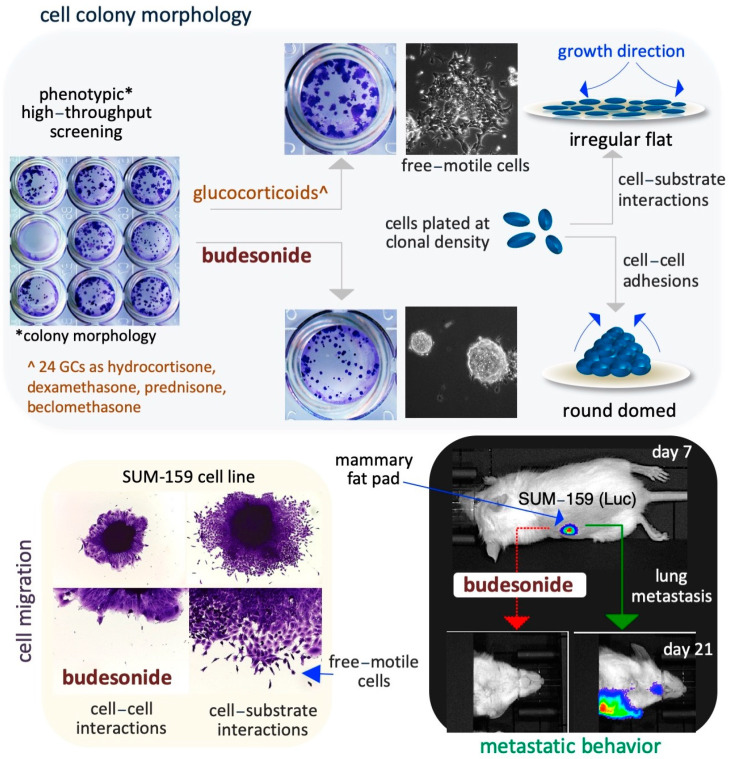
Schematic representation of the inhibitory effect of BUD on stem and cancer cell plasticity. A phenotype-based HTS reveals that BUD, unlike the 24 other examined GCs, is a potent modulator of the cell colony morphology in ESCs (upper images). With regard to the specific mechanisms, BUD appears to play a role in maintaining cell–cell adhesive interactions by inducing the generation of highly compacted cell colonies (upper right diagrams). In addition, BUD inhibits the migration of SUIM-159 breast cancer cells favoring cell–cell adhesions (bottom left images). Similar results are obtained by assaying A549 lung cancer cells. Moreover, in an in vitro mouse model of breast cancer, BUD is found to reduce lung metastasis from primary mammary tumors (bottom right images).

Cell plasticity contributes to tissue development and homeostasis in multicellular organisms and to the progression of pathologies such as metastatic cancer and organ fibrosis. Emerging evidence has revealed that BUD reduces cell plasticity (that is, motility and invasiveness) in stem and cancer cells, including lung, breast, and pancreatic cancer cells [96] (Figure 3). The next few subsections focus on the BUD inhibition of cell plasticity and the related mechanisms.

### 4.1. Lung Cancer Cells

In tumor spheroid and Boyden chambers assays, BUD (10–20 μM) has been found to inhibit the migration, but not the proliferation, of human A549 cells (pulmonary alveolar type II), a cell model of lung cancer [96]. A comprehensive understanding of the molecular mechanisms altered by BUD in A549 cells is lacking, but there is some evidence to suggest a link between motile behavior/mesenchymal phenotype and collagen synthesis. That is, BUD has been found to inhibit collagen synthesis and maturation at the RNA and protein level [96]. Moreover, the researchers generated A549-derived cell lines expressing low levels of the 4-prolyl-collagen hydroxylation (P4HA) enzyme (*P4HA2^KD^*) through lentiviral infection of shRNAs [96]. Notably, reduced expression of the P4HA2 enzyme was found to significantly reduce both hydroxylated collagen accumulation (~10 times) and cell migration (from 80% to 20%), without altering the proliferation of A549 cells [96]. While it is unclear how collagen synthesis/hydroxylation influences cell migration, a vitamin C (VitC)-dependent modification of the epigenetic landscape appears to be involved. From these findings, it could be speculated that BUD potentially exerts anti-metastatic effects via the suppression of collagen production/hydroxylation; further, its mechanisms could involve VitC-dependent epigenetic modifications, which will be discussed in detail later on (see Section 6.2).

### 4.2. Breast Cancer Cells

BUD was found to block cell invasion and metastatic dissemination of the triple-negative breast cancer cell line SUM159 under both in vitro (in 3D organotypic culture) and in vivo (in an orthotopically injected mouse model) conditions, as described below. SUM159 cells are phenotypically metastable/heterogenous and are able to undergo collective invasion, a typical feature of human breast cancer cells [96]. BUD reduces the collective invasive ability of SUM159 spheroids (Figure 3) and increases colony compaction and the formation of round-shaped colonies (circularity) in a dose-dependent manner, without affecting cell proliferation [96]. The authors confirmed the in vitro results in an experimental metastatic murine model that was generated by the injection of luciferase-expressing SUM159 cells into the mammary fat pad of immunocompromised mice (Figure 3). BUD treatment of these mice resulted in a reduction in the size and volume of the primary tumor and their histology, including stromal collagenolytic organization, display a less organized collagen-surrounding extracellular matrix. Remarkably, BUD abrogates the formation of metastatic foci in the lungs (Figure 3) [96]. Overall, these findings imply that BUD reduces collagen production/deposition and hampers the mesenchymal transition of tumor cells, ultimately hindering the metastatic dissemination of breast cancer.

### 4.3. Pancreatic Cancer Cells

In Boyden chamber and Cy3-gelatin invadopodia assays (2D growth settings), BUD (supplemented at 20 μM) was found to modify the behavior of pancreatic adenocarcinoma (PDAC) cells by reducing their mesenchymal motile/invasive features [97]. Unlike BUD, other glucocorticoids, such as fluticasone, dexamethasone, and hydrocortisone, were unable to modify the phenotype of various 2D-cultured PDAC cell lines when used at a similar concentration range. Although higher concentrations of GCs were not assessed, the analysis of a cell line expressing low levels of GR (*NR3C1*-knockdown PDAC cells) excluded the role of the hormone–GR axis in the BUD-induced epithelization of PDAC cells [97]. In a more physiological 3D environment, such as 3D floating spheroids or organotypic cultures (Matrigel), PDAC cells were found to undergo global metabolic reprogramming involving energy metabolism. As a result of these changes, PDAC cells became increasingly dependent on glycolysis to obtain energy and were sensitized to the unexpected anti-proliferative action of GCs, including BUD, dexamethasone, and hydrocortisone. Further, BUD was found to inhibit PDAC cell proliferation even at low (at the nM scale) concentrations, in a GR-dependent manner and, at least in part, through the induction of the tumor suppressor Cyclin-Dependent Kinase Inhibitor 1C (CDKN1C). Remarkably, a lower occurrence of PDAC has been described in individuals with severe and long-standing asthma [98], and a first-line therapy to treat asthma is budesonide [3,99].

## 5. Budesonide-Mediated Stabilization of Cell–Cell Interactions

**Figure 4:**
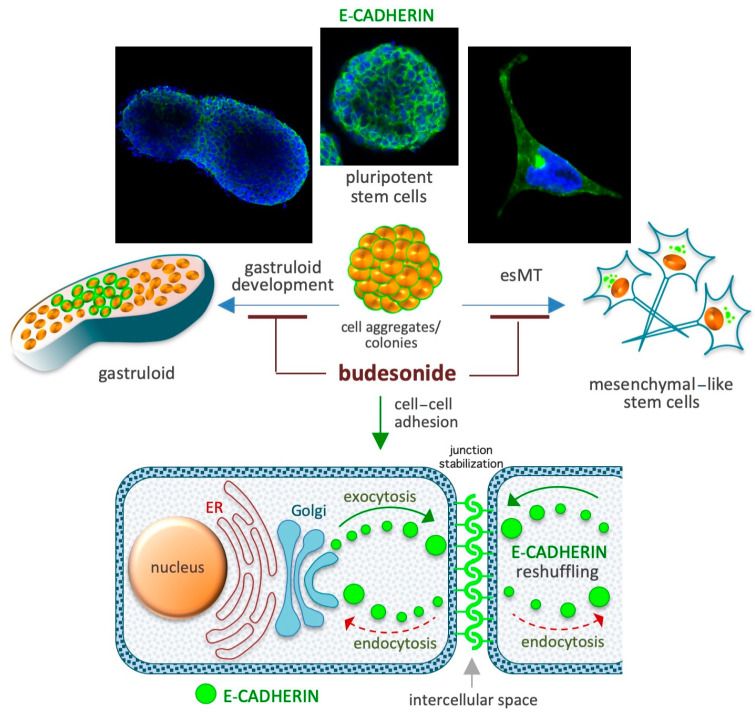
Schematic depicting the role of BUD in the maintenance of high levels of the adhesive protein E-cadherin at the cell–cell interface. Spontaneous gastruloid development and proline-induced embryonic-stem-to-mesenchymal transition (esMT) are depicted in the upper diagrams. A similar role of BUD involving its effect on E-cadherin expression has been described in chronic IDs involving the gastroenteric and airway mucosa. The diagram at the bottom depicts E-cadherin-mediated cell junction stabilization based on the inhibition of its intercellular-to-intracellular delocalization (reshuffling).

Adhesive cell–cell interactions are important players in cell behavior and are dependent on proteins involved in the formation of tight and adherens junctions, such as claudins and cadherins. For instance, in stem cells, the induction of the CDH1 gene (which codes for E-cadherin) is crucial for maintaining the naïve/ground state of pluripotency, whereas its downregulation is essential for pluripotency exit, cell differentiation, and embryo/tissue development [100,101,102,103,104,105]. E-cadherin is also required to maintain epithelial integrity/barrier activity in different types of mucosal tissues [102]. The effect of BUD on cell plasticity is mediated by its effects on these proteins, as emerging evidence suggests that BUD preserves high levels of expression of adhesive proteins at the cell–cell interfaces in different stem and somatic cell models (Figure 4). In particular, the adhesive protein E-cadherin has been implicated in these effects of BUD on cell–cell adhesion in the context of both stem cell pluripotency and the preservation of mucosal structure and integrity, as described in the next few subsections.

### 5.1. Preservation of the Naïve Ground State in Stem Cells

A cocktail containing the cytokines LIF, CHIR99021, and PD032590 is used to maintain the naïve ground state of pluripotency in mouse ESCs [106]. When naïve-state ESCs are incubated for 3–5 days in cell proliferation medium, they form highly compacted E-cadherin-expressing cell colonies displaying a typical round 3D/domed shape. When the naïve state-inducing factors are removed from the medium, the stem cells undergo spontaneous differentiation (pluripotency exit), display altered growth behavior, and generate irregular 2D/flat-shaped cell colonies that are surrounded by free-motile cells (Figure 4). E-cadherin, which is essential for the preservation of both aggregation propensity and naïve pluripotency [107], is downregulated during spontaneous stem cell differentiation. BUD (in a 5–20 μM concentration range) and a few BUD analogs have been found to delay pluripotency exit at a morphological and molecular level by stabilizing E-cadherin at the plasma membrane. Of note, BUD alone, in the absence of naïve state-inducing factors, preserves both high levels of expression of pluripotency-related transcription factors (e.g., OCT4, Nanog, and SOX2) and a naïve-like dome-shaped cell colony morphology. Thus, BUD maintains strong cell–cell interactions among ESCs through its effects on E-cadherin and pluripotency-related transcription factors [108,109].

### 5.2. Naïve-to-Primed Transition in Pluripotent Stem Cells

In the presence of the non-essential amino acid proline (at a concentration range of 150–250 μM) and low levels of ascorbic acid (VitC), mouse ESCs are captured in an early primed state of pluripotency [110,111,112,113,114]. Proline supplementation induces E-cadherin delocalization (Figure 4) from the cytoplasmic membrane to trans-Golgi vesicles in the cell cytoplasm and, thus, cell disconnection/loosening/detachment [110,111]. An unbiased phenotype-based HTS of ~1200 FDA-approved drugs revealed that BUD was a potent inhibitor of proline action in ESCs [96]. Unlike the 24 other glucocorticoids that were screened (including hydrocortisone and dexamethasone), BUD (when administered at a dose of 10 μM) was found to stabilize E-cadherin in the intercellular space and, thereby, prevent the effect of proline [96], which relies on the modulation of several signaling pathways including the AAR/ATF4 (amino acid stress response), Wingless and Int-1 (WNT/β-catenin), fibroblast growth factor/extracellular signal-regulated kinase (FGF/ERK), and TGFβ pathways [113,115]. Indeed, similar to the effects of BUD treatment, the overexpression of activating transcription factor 4 (ATF4) and supplementation with either CHIR99021 (a WNT agonist) or PD0325901 (a MEK/ERK inhibitor) were also found to inhibit the effects of proline [96,110,113].

### 5.3. Gastruloid Development

Gastruloids are early embryo-like structures that develop from 3D aggregates of ESCs and establish the axial organization of post-implantation embryos [116,117,118]. The emergence of spatially ordered structures (that are polarized and elongated) from homogenous/spherical cell aggregates is reliant on an initial event called symmetry breaking, which refers to the process by which homogeneity is broken. BUD (at a dose of 5–20 μM) prevents, in a GR-independent manner, symmetry breaking and gastruloid elongation. Indeed, BUD was found to hamper pluripotency exit by altering the expression of genes related to cell migration, anterior–posterior axis formation, and WNT signaling (Figure 4). In the presence of BUD, the E-cadherin protein persists at the cell–cell interface, and the cell aggregates maintain, even after 5 days of incubation in the absence of naïve state-inducing factors, a compacted spherical geometry, and the expression of pluripotency markers [108]. BUD analogs were found to result in similar gastruloid inhibition, even at a 10-times lower concentration [109]. In the absence of WNT activation, E-cadherin expression is maintained, and a major fraction of the aggregates retain a spheroidal shape [119]. Of note, in vivo gastrulation in a mouse model was found to be dependent on cadherin regulation [120]. These findings imply that the inhibitory effects of BUD on gastruloid development involves the modulation of cadherin localization and WNT signaling.

### 5.4. Gastroenteric Mucosa

E-cadherin is essential for preservation of the structure and function of the intestinal mucosa [121]. Accordingly, genes involved in mucosal barrier function, namely, *CDH1* (E-cadherin), *ECM1* (extracellular matrix protein 1), and *LMNB1* (extracellular matrix glycoprotein laminin B1), are associated with the risk of UC in humans [122]. Importantly, the membrane-to-cytoplasmic delocalization of E-cadherin due to *CDH1* polymorphisms is considered one of the main causes of epithelial failure in Crohn’s disease [123]. Accordingly, monoclonal antibodies able to stabilize the E-cadherin complex were found to strengthen cell–cell adhesions [124] and impede the progression of inflammatory bowel disease in mice [121]. Thus, the induction and stabilization of E-cadherin in the plasma membrane is a potential strategy to manage chronic colitis [121]. Of note, in a mouse model of colitis induced by the intrarectal acetic acid challenge, BUD treatment was found to normalize E-cadherin expression and cell–cell adhesion [125]. Thus, the therapeutic effect of BUD in gastroenteric mucosal diseases may involve re-epithelialization mechanisms exerted via the normalization of E-cadherin expression and cell–cell adhesion.

### 5.5. Airway Mucosa

E-cadherin is essential for preserving the structure and function of the airway mucosa [126]. Indeed, the airway epithelium in asthma patients is characterized by the reduced expression of many intercellular adhesion molecules, including E-cadherin, which results in impaired barrier function and increased invasion by external pathogens and allergens [127,128,129].

## 6. Budesonide-Mediated Inhibition of Collagen Deposition and Modulation of the Epigenetic Landscape

**Figure 5:**
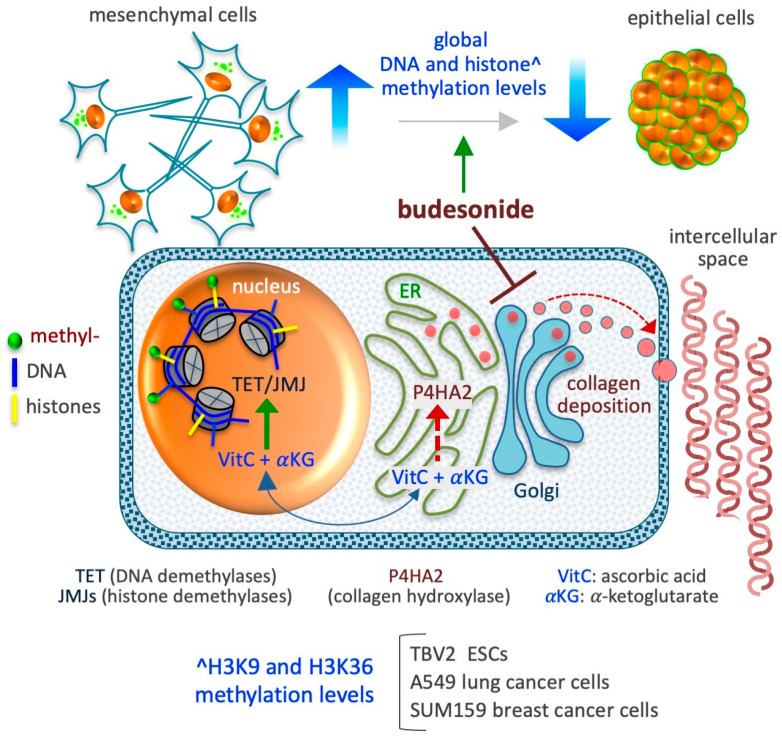
Schematic depicting the impact of BUD on the epigenetic landscape of stem and cancer cells. BUD decreases DNA and histone methylation levels and induces mesenchymal-to-epithelial transition. The model presented at the bottom depicts the compartmentalization of two metabolites, ascorbic acid (VitC) and alpha-ketoglutarate (αKG), which are essential co-factors/substrates for the activity of dioxygenase enzymes involved in collagen hydroxylation (as prolyl 4-hydroxylase subunit alpha 2/P4HA2) in the cytoplasm (endoplasmic reticulum/ER) and in DNA and histone hydroxylation/demethylation (as ten-eleven translocation/TET and Jumonji/JMJ enzymes) in the nucleus. As proposed in D’Aniello et al. [96], when collagen synthesis is inhibited in the ER, the availability of VitC and αKG increases in the nucleus, thus favoring the activity of nuclear DNA and histone demethylases.

The impact of BUD on the epigenetic profile of cells is regaining interest as a result of an ongoing clinical trial managed by the University of British Columbia (trial registration no. NCT04342039) in Canada entitled “Epigenetic Health Benefits of BUD” that is due for completion in December 2024. This pilot study aims to test the ability of BUD to reverse the epigenetic modifications induced by allergens and pollutants in patients with nasal rhinitis, an allergic inflammatory disease. Past research has demonstrated these epigenetic modifications in the context of various cancers and inflammatory conditions, as well as stem cells. Here, we present an overview of the current knowledge of how BUD may impact the epigenetic profile of stem and cancer cells (Figure 5).

### 6.1. DNA Methylation

In stem cells subject to a high-proline/low-VitC regimen, the level of 5-hydroxymethylcytosine, which prompts DNA demethylation, rapidly decreases [110]. However, this reduction does not occur in ESCs deficient in the collagen hydroxylation/maturation enzyme P4HA. Importantly, BUD mimicked the antagonistic effect of a high-VitC regimen and prevented both proline-dependent esMT (see Section 5.2) and the increase in DNA methylation levels. Early studies revealed the chemopreventive action of BUD in lung carcinogenesis and a BUD-induced increase in DNA methylation in lung cancer cells. BUD was found to delay the appearance of lung tumors or their progression to carcinoma in mouse and human cancers [130,131,132,133,134,135,136,137,138]. For instance, a double-blind randomized trial (trial no. NCT00321893) shows that the inhalation of BUD (800 µg twice daily for 1 year) reduces the size of non-solid lung nodules in high-risk humans, even over 5 years of follow up [130]. Further, in mouse models of lung cancer, BUD treatment (2 mg/kg administered orally as part of the diet) was found to reduce the size of solid lung adenomas induced by vinyl carbamate (administered intraperitoneally at a dose of 16 mg/kg) [139]. Although the mechanisms underlying these actions of BUD are not entirely clear, it is speculated that changes in the epigenetic profile of the cells may be involved. Indeed, the intake of BUD (added to the diet at a dose of 0.6 to 2.4 mg/kg) for 7 days has been found to increase the total DNA methylation level (based on dot blot analysis) of lung tumors in mice [136,140]. In particular, BUD induces the methylation of CpG islands associated with two tumor-related genes, namely, Insulin-like Growth Factor 2 (IGF-2) and the Cellular Myelocytomatosis (c-MYC) oncogene [136,140]. In the AA8 cell line (hamster ovary fibroblasts) exposed (24 h) to a high-dose BUD regimen (10–70 μM), the level of global DNA methylation (based on measurements of 5-methycitosine levels by high-performance liquid chromatography) was found to increase in a dose-dependent manner [58]. However, the pathways via which BUD influences DNA methylation levels remain unclear. A recent review indicated that BUD may function as an activator of DNA methyltransferases [141], but experimental evidence of a direct interaction between BUD and DNA methyltransferases is lacking. In the future, genome-wide next-generation sequencing approaches could be applied to analyze how the DNA methylation landscape responds to BUD.

### 6.2. Histone Methylation

The increased availability of proline to ESCs leads to an abrupt increase in collagen synthesis, which drives the cells to acquire a mesenchymal invasive behavior—a transition associated with a robust increment in the methylation levels of H3K9 and H3K36 [110]. A genome-wide analysis of these changes has revealed the involvement of more than 15,000 H3K9 methylation sites, mainly located in noncoding intergenic regions of the genome, with the greatest enrichment of H3K9me3 sites observed in the constitutive heterochromatin, suggestive of a shift toward more dense structures in the nucleus [110]. An increment in the methylation level in more than 8500 H3K36 methylation sites, mainly at noncoding regions, is also indicative of heterochromatin modulation. Of note, the administration of either BUD (at a dose of 10 µM) or VitC (at a dose of 50 µg/mL) has been found to revert the epigenetic effects of proline by reducing the histone methylation levels at the basal level of untreated cells. In A549 lung cancer cells, BUD was found to reduce global histone (H3K9me2/me3, H3K36me3, H3K27me3, and H3K4me3 methylation) methylation levels by up to 50% [96]. Further, BUD was also found to reduce expression of the P4HA2 enzyme (in a P4HA2^KD^ cell line), consequently resulting in a reduction in the level of hydroxylated collagens, and to reduce the histone methylation levels (by up to 60%), without altering the proliferation of A549 cells. In SUM159 breast cancer cells, BUD prevents the mesenchymalization of the cells under both in vitro (in 3D organotypic culture) and in vivo (in orthotopically injected mouse models) conditions [96], thus reducing global histone (H3K9me2/me3, H3K36me3, H3K27me3, and H3K4me3methylation) methylation levels [96]. A preliminary quantitative measurement reveals that BUD (supplemented at a concentration of 20 µM) also reduces the methylation level of other lysine residues of histone H3, including K4 (me1/me2/me3), K9 (me1/me2/me3), K18me1, K23me1, K36m3, and K79 (me1/me2) in SUM159 breast cancer cells (our unpublished data). Together, these data support the existence of a metabolic interplay that links collagen hydroxylation in the endoplasmic reticulum (ER) and DNA/histone hydroxylation (demethylation) in the nucleus [112,142,143], mediated by changes in the availability of ascorbic acid (VitC) in the different cellular compartments. Indeed, VitC acts as a cofactor for enzymes involved in both collagen hydroxylation and histone demethylation [143]. In brief, when collagen synthesis abruptly increases in the ER, VitC is consumed at high rates for the hydroxylation of nascent collagens, reducing the availability of reduced VitC for DNA and histone demethylation in the nucleus (Figure 5). In such conditions, the administration of BUD could influence epigenetic modifications in the form of histone methylation.

### 6.3. Histone Deacetylation

Inhaled corticosteroids suppress mucosa inflammation in patients with chronic IDs such as asthma by downregulating the expression of inflammatory genes. One of the mechanisms that is activated to switch off the transcription of inflammatory genes is a reduction in the histone acetylation levels via the recruitment of histone deacetylase 2 (HDAC2) [144]. Accordingly, it has been reported that steroid resistance, which is the inability of steroids to reduce lung inflammation, is related to the Nrf2-HDAC2 axis [145]. For instance, BUD inhibits lipopolysaccharide-induced lung inflammation in normal mice but not in mice lacking HDAC2 expression [145]. BUD-mediated DNA hypermethylation could induce the binding of histone deacetylases (HDAC enzymes), thereby reducing histone acetylation levels, increasing chromatin condensation, and, thus, inhibiting gene expression [139].

## 7. Budesonide-Mediated Inhibition of Replication of SARS-CoV-2 and Single-Stranded (+)RNA Virus

**Figure 6:**
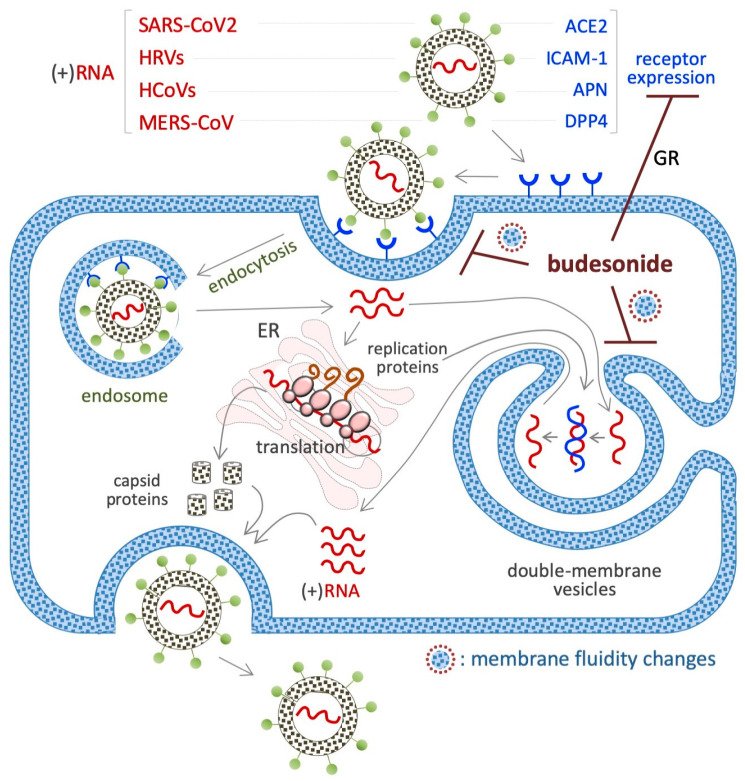
Schematic showing the inhibitory effect of BUD on the infection/replication of (+)RNA viruses. The membrane receptors of each virus are indicated in the upper part of the diagram. BUD inhibits the infection/replication of these viruses by reducing the expression of their receptors (angiotensin-converting enzyme-2/ACE2 for SARS-CoV2 and intercellular adhesion molecule 1/ICAM-1 for HRV) or interfering with the trafficking/fluidity of the membrane complexes that are necessary for the endocytosis (endosome) and/or the replication (double membrane vesicles) of the virus.

BUD has been found to inhibit infection with and/or the replication of various single-stranded (+ssRNA or (+)RNA) human-infecting RNA viruses, including severe acute respiratory syndrome coronavirus 2 (SARS-CoV-2) [146], rhinovirus (HRV) [147], human coronavirus (HCoV) [148], and Middle East respiratory syndrome (MERS) [149] (Figure 6), as described in the next few subsections.

### 7.1. SARS-CoV2 (+)RNA Virus

A beneficial impact of BUD treatment on early COVID-19 disease emerged from several randomized trials and meta-analysis. Through its action as an anti-inflammatory glucocorticoid, BUD was found to prevent the cytokine storm that is observed in some COVID-19 patients, i.e., an excessive immune response associated with multi-organ failure and fatalities [150]. In vitro experiments have shown that BUD inhibits SARS-CoV-2 infection of cultured VeroE6 cells in a dose-dependent manner (when administered at doses ranging from 0.1 to 25 µM) [146] and is effective against several variants of the virus at an IC_50_ of 5–20 μM [146]. With regard to the related mechanisms, BUD was found to reduce the expression of the SARS-CoV-2 receptor angiotensin-converting enzyme-2 (ACE2) in a type I interferon-beta (IFN-β)-dependent manner (Figure 6) [151]. This might suppress the entry of the SARS-CoV-2 virus into cells. Of note, ACE2 also regulates the membrane trafficking of the neutral amino acid transporter SLC6A19, a sodium-dependent and chloride-independent proline transporter. This observation could explain, at least in part, how BUD counteracts the ability of supplemental proline to induce esMT in mouse ESCs (see Section 4.1) [111,152].

### 7.2. HRVs (+)RNA Virus

HRVs cause the common cold. A screening of 800 FDA-approved drugs revealed that BUD inhibits HRV-mediated cytotoxicity in HeLa cells [147]. BUD (10 nM) was also found to inhibit HRV infection in primary cultures of human tracheal epithelial cells [153]. A reduction in the expression of the intercellular adhesion molecule-1 (ICAM-1) gene, coding for the HRV receptor, could contribute to this action of BUD. Further, as a classical glucocorticoid, BUD (1 μM) prevents the HRV-mediated induction of the potent inflammatory cytokine pro-interleukin-1beta (IL-1β), in mouse bone marrow-derived macrophages [147], as well as the cytoplasm-to-nuclear translocation of the nuclear factor kappa-B (NF-kB) and the expression of IL-1β, IL-6, and IL-8, in human tracheal epithelial cells [153].

### 7.3. HCoV (+)RNA Virus

This virus infects host cells by binding to the aminopeptidase N (APN) receptor, and HCoV-229E invades cells via the APN receptor–endosome axis. BUD, when combined with glycopyrronium and formoterol, was found to inhibit HCoV-229E (the etiological agent of common cold) infection of primary human nasal and tracheal epithelial cells [148]. While the molecular mechanism underlying HCoV inhibition remains unknown, the downregulation of APN expression and inhibition of endosomal function could be involved.

### 7.4. MERS-CoV (+)RNA Virus

The MERS-CoV virus enters its host cell by binding to the dipeptidyl peptidase IV (DPP4) receptor [154]. Ciclesonide, a steroid with a pentacyclo structure involving positions 16 and 17 of the steroid backbone, inhibits the replication of MERS-CoV and SARS-CoV-2 in VeroE6 cells, and it was found to be more efficient than BUD [149]. In addition, ciclesonide and BUD could target the viral replication–transcription complex in differentiated human bronchial tracheal epithelial cells. However, under similar conditions, cortisone, dexamethasone, and fluticasone are unable to inhibit the replication of this virus.

### 7.5. Viral Mimetic dsRNA

BUD was tested in a cellular model of virus-induced airway epithelial barrier disruption, i.e., a monolayer of human bronchial epithelial cells (16HBE) exposed to dsRNA/poly(l:C) [155]. The results showed that BUD treatment limited trans-epithelial electrical resistance and small molecule permeability (according to the results of 4 kDa FITC-dextran flux experiments) [156]. In addition, BUD was also found to be effective against poly(l:C)-induced airway barrier disruption in the lungs of mice, independent of its anti-inflammatory activity [156]. It has been observed that BUD can be incorporated into cell membranes [23] and, such as cholesterol, can alter the fluidity of cell membranes. Remarkably, the generation of intracellular double-membrane vesicles is essential for the replication of (+)RNA human-infecting RNA viruses (Figure 6).

## 8. Concluding Remarks and Future Prospects

As described in the previous sections, based on recent evidence, it is now clear that BUD has an impact on several different cell types other than immune cells. However, apart from GR, the receptor/molecule(s) targeted by BUD remain unknown. BUD, like cholesterol and sterols, is incorporated into cell membranes [23], and a significant fraction (~80%,) is rapidly converted (~20 min) into lipophilic fatty acid esters (through esterification with palmitate or oleate at the C-21 position) [51,157,158,159,160,161]. Since fatty acid esters can be hydrolyzed (reversible esterification) by intracellular lipases, BUD conjugates are considered an intracellular pool/reserve of the drug that are useful for extending its anti-inflammatory action [160]. However, the impact of BUD, or BUD conjugates, on plasma membrane-related activities, i.e., signaling, cell permeability, endosome recycling, and cytoskeleton conformation/cell nuclear morphology, remains unexplored. Of note, BUD could influence the composition/biophysical properties (that is, fluidness and stiffness) of cholesterol- and sphingolipid-enriched raft microdomains, which are key players in the modulation of many relevant cell signaling pathways. Indeed, the effect of BUD-membrane interaction on cell properties deserves further investigation. Specifically, a comparison of the effect of BUD on the transcriptome, epigenome, and metabolome of GR+ and GR- cell lines could be very useful to understand which processes are regulated by BUD independently of GR.

Emerging evidence indicates that the cellular/molecular process targeted by BUD to efficiently reduce the symptoms/progression of IDs are complex and still not fully understood. Indeed, the mucosa of different gastroenteric (esophagus, stomach, and gut) and airway (larynx, bronchi, and lung) tracts differ in terms of their histological organization and the types and number of epithelial layers. However, a common feature of different mucosal tissue is the generation and maintenance of robust cell–cell interactions, which ensures barrier activity, i.e., the isolation of internal organs from external microbes and/or toxicants (chemicals, allergens), as well as the finely regulated exchange of ions and water. Here, we propose that reduction in the intercellular adhesive contacts is a triggering event in ID pathogenesis, and this is followed by the infiltration of microbe/toxicants and, eventually, the activation and transmigration of immune cells (eosinophils, lymphocyte, and macrophages) into the mucosa. In addition, as demonstrated in the literature, the deregulation of epithelial-related genes and extracellular matrix remodeling are associated with different IDs. In this scenario, BUD could support the “re-epithelization” (mucosa regeneration) of the swollen mucosa in at least two different ways: (1) by inhibiting the activation/transmigration of immune cells and (2) by modulating the expression/activity of the genes involved in cell–cell interactions, thus favoring epithelial stabilization and the reestablishment of its barrier activity.

Recent studies have highlighted an association between the peculiar epithelization action of BUD and the stabilization of the adhesive protein E-cadherin in the intercellular spaces. Indeed, three models of pluripotency exit in ESCs, including gastruloid development and embryonic stem-to-mesenchymal transition, suggest the existence of a finely regulated BUD–E-cadherin–pluripotency axis. E-cadherin expression is induced by activation of the WNT signaling pathway, and a WNT agonist is required to induce gastruloid elongation/development. This could mean that BUD, directly or indirectly, controls the cell membrane localization and/or the adhesive activity of E-cadherin. A notable finding in this regard is that E-cadherin expression is induced and can replace OCT4 during the reprogramming of somatic cells towards induced pluripotent stem cells [162]. However, it is unclear whether BUD has a putative beneficial effect on the reprogramming of somatic cells, and this is a topic that warrants investigation in the future.

E-cadherin-mediated adhesive cell–cell interactions are also important in cancer cell biology. For instance, E-cadherin is required to generate adherent clusters of cancer cells that undergo collective cell migration [163,164]. Moreover, E-cadherin delocalization (from the plasma membrane to the cytoplasm) is associated with the acquisition of a mesenchymal/free motile phenotype [165,166]. In line with this, BUD has been found to stabilize E-cadherin-mediated cell–cell interactions and reduce mesenchymal features in breast, lung, and pancreatic cancer cells. Additional in-depth studies would be required to understand the pathways via which BUD targets E-cadherin signaling. In this regard, cell–cell interactions can be strengthened by exploiting the increased binding affinity of cadherin clusters as compared with cadherin monomers [167]. Further, lipid rafts and lipid raft-associated proteins could be crucial for cadherin cluster organization. Thus, it might be interesting to investigate the existence of a BUD-raft microdomain–E-cadherin cluster axis. We speculate that a similar mechanism action may also be involved in the BUD-mediated healing of inflamed mucosa and contribute to the remission of IDs. In addition, BUD-derived bioactive compounds, which would be able to induce similar effects but with higher potency, are possibly potential drug candidates to treat a broad spectrum of diseases involving cell mesenchymalization such as cancer progression/metastasis.

The molecular mechanisms underlying the BUD-mediated inhibition of virus infection/replication remains unknown. Although the viruses that BUD inhibits are all positive-sense single-stranded RNA viruses, the extracellular receptors involved are different (ACE2, APN, ICAM-1, and DPP4). Despite this, the involvement of BUD–cytoplasm membrane interactions in virus-receptor recycling (endosomal traffic) cannot be excluded. Since (+)RNA virus genomes have the ability to act as messenger RNA, their genomes are directly translated into proteins by host ribosomes. Once viral proteins are produced inside the host, they recruit the RNA to produce viral replication complexes. In this way, viral replication continues through double-stranded RNA intermediates. The inhibition of viral replication when cholesterol trafficking is blocked suggests that these dynamic changes in trafficking are functionally significant for the virus. Thus, BUD could alter membrane fluidity/proprieties that are required for efficient viral replication, and this deserves further investigation.

In conclusion, the multi-pronged effects of BUD in various disease contexts, including IDs, cancers, and viral infections, make it a potent therapeutic agent that could have immense potential if used in conjunction with other drugs. Future in-depth research into its mechanisms could provide a richer understanding of the molecular landscape of these conditions and shed light on more effective therapeutic strategies, including BUD.

## Abbreviations

The following abbreviations are used in this manuscript:

ACE2	angiotensin-converting enzyme-2
αKG	alpha-ketoglutarate
ASM	airway smooth muscle
ATF4	activating transcription factor 4
BUD	budesonide
cAMP	cyclic adenosine monophosphate
CDH1	Cadherin 1
CDKN1C	Cyclin-Dependent Kinase Inhibitor 1C
COX-2	cyclooxygenase-2
c-MYC	Cellular Myelocytomatosis
ECM1	extracellular matrix protein 1
EGF	extracellular signal-regulated kinase
EoE	eosinophilic esophagitis
EMT	epithelial-to-mesenchymal transition
ER	endoplasmic reticulum
ESCs	embryonic stem cells
esMT	embryonic-stem-to-mesenchymal transition
FGF	fibroblast growth factor
GCs	Glucocorticoids
GPCR	G protein-coupled receptor Gα
GR	glucocorticoid receptor
HCoV	human coronavirus
HDAC	histone deacetylase
5hMC	5-hydroxymethylcytosine
HRVs	human rhinoviruses
HTS	high-throughput screening
ICAM-1	intercellular adhesion molecule-1
IDs	inflammatory diseases
IGF-2	Insulin-like Growth Factor 2
IFN-β	type I interferon-beta
IL-1β	Interleukin-1 β
IL-6	Interleukin-6
IL-8	Interleukin-8
JMJ	Jumonji
LIF	leukemia inhibitory factor
LMNB1	extracellular matrix glycoprotein laminin B1
MC	microscopic colitis
MERS	Middle East respiratory syndrome
MPO	myeloperoxidase
N3CR1	nuclear receptor subfamily 3 group C member 1
NF-kB	nuclear factor kappa-B
Nrf2	nuclear factor erythroid 2-related factor
PDAC	pancreatic adenocarcinoma
P4HA	prolyl-collagen hydroxylation
P450/CYP3A	cytochrome P4503A
SARS-CoV-2	severe acute respiratory syndrome coronavirus 2
TET	ten-eleven translocation
TGFβ1	transforming growth factor-β1
TGM1_3	transglutaminase enzymes 1 and 3
TNF	tumor necrosis factor
UC	ulcerative colitis
VitC	vitamin C
WNT	Wingless and Int-1
